# Grain Yield and Water Use Efficiency in Extremely-Late Sown Winter Wheat Cultivars under Two Irrigation Regimes in the North China Plain

**DOI:** 10.1371/journal.pone.0153695

**Published:** 2016-04-21

**Authors:** Bin Wang, Yinghua Zhang, Baozhen Hao, Xuexin Xu, Zhigan Zhao, Zhimin Wang, Qingwu Xue

**Affiliations:** 1 College of Agronomy, China Agricultural University, Beijing, 100193, China; 2 Texas A&M AgriLife Research and Extension Center at Amarillo, Amarillo, Texas, 79106, United States of America; 3 School of Science and Technology, Xinxiang University, Xinxiang, Henan, 453003, China; Institute of Genetics and Developmental Biology, CHINA

## Abstract

Wheat production is threatened by water shortages and groundwater over-draft in the North China Plain (NCP). In recent years, winter wheat has been increasingly sown extremely late in early to mid-November after harvesting cotton or pepper. To improve water use efficiency (WUE) and guide the extremely late sowing practices, a 3-year field experiment was conducted under two irrigation regimes (W1, one-irrigation, 75 mm at jointing; W2, two-irrigation, 75 mm at jointing and 75 mm at anthesis) in 3 cultivars differing in spike size (HS4399, small spike; JM22, medium spike; WM8, large spike). Wheat was sown in early to mid-November at a high seeding rate of 800–850 seeds m^−2^. Average yields of 7.42 t ha^−1^ and WUE of 1.84 kg m^−3^ were achieved with an average seasonal evapotranspiration (ET) of 404 mm. Compared with W2, wheat under W1 did not have yield penalty in 2 of 3 years, and had 7.9% lower seasonal ET and 7.5% higher WUE. The higher WUE and stable yield under W1 was associated with higher 1000-grain weight (TGW) and harvest index (HI). Among the 3 cultivars, JM22 had 5.9%–8.9% higher yield and 4.2%–9.3% higher WUE than WM8 and HS4399. The higher yield in JM22 was attributed mainly to higher HI and TGW due to increased post-anthesis biomass and deeper seasonal soil water extraction. In conclusion, one-irrigation with a medium-sized spike cultivar JM22 could be a useful strategy to maintain yield and high WUE in extremely late-sown winter wheat at a high seeding rate in the NCP.

## Introduction

The North China Plain (NCP) is one of the most important wheat production areas in China, with 60% of the national wheat production [1]. With a monsoon climate, the winter is cold and dry and > 70% of the annual precipitation falls in the summer months (July through September) [2]. In conventional wheat production, early sowing (late September–early October) with sufficient soil moisture at sowing are required for strong seedling growth and development. Generally, 1–2 times irrigation before winter is necessary to meet water demand for overwintering, and 2–3 times irrigation is applied in spring to achieve high yield. However, water shortage is an important factor limiting wheat production in this area. Irrigation in wheat production accounts for 70% of the total agricultural water use, with 64% of the water from groundwater [3, 4]. Overdraft of groundwater has resulted in a rapid decline in the groundwater table, threatening sustainable agricultural development in the region [1]. Recently, a water-saving farming system was developed based on that moderately late sowing to decrease main stem leaf stage from 6–7 to 4–5 Huan stage [5] and reduce evapotranspiration (ET) before winter. At the meantime, two irrigation regimes (about 75 mm at jointing and 75 mm at anthesis) can be used to increase water use efficiency (WUE) and maintain yield [6–8]. Nevertheless, groundwater overdraft problems are still serious under the optimized irrigation regimes, raising the concern of both the public and government [9]. To stabilize the groundwater table, it is urgent to explore further irrigation reduction while maintain yield and further increase WUE.

Winter wheat in the NCP is sown mainly in early-mid October after summer maize or in late October after harvesting cotton or other preceding crops. With the cropping system adjustment and development of economic crops, pepper with late harvest has become more and more popular in the recent years. For example, Heibei province is one of largest cotton and pepper production areas in China, with an area of > 68×10^4^ ha cotton and > 6.7×10^4^ ha pepper, respectively [10, 11]. Consequently, the sowing date in winter wheat has been delayed with the late harvest of preceding crops such as pepper. In addition, other factors such as lack of adequate soil moisture, insufficient labor, or no necessary machinery also contributed to delaying wheat sowing. In particular, the sowing date of winter wheat after pepper has been delayed to early-mid November. Wheat plants sown extremely late normally at less than 1.0 Haun stage [5] before wintering and have no tiller development during growing season. Currently, little information is available for the extremely-late sown winter wheat management. In general, late sowing decreases early growth stages and the seasonal ET in wheat [12]. Therefore, information about the performance of extremely-late sown winter wheat with minimum irrigation could provide important guidance in wheat production and conserve groundwater resources.

Previous studies have shown that late sowing reduced yield mainly because of decreased spikes m^−2^ [13, 14], grains spike^−1^ [12, 15] or 1000-grain weight (TGW) [12]. Wheat yield is determined largely by population size (spikes m^−2^) and spike quality (spike fertility) during wheat growing season [16]. A high seeding rate is required to develop sufficient numbers of spikes in late-sown winter wheat because of little or no tiller development. Moreover, selecting suitable cultivars is critical for high yield. Thus, understanding characteristics of biomass accumulation and partitioning over time in different cultivars under minimum irrigation is essential for improving both yield and WUE in extremely late-sown wheat.

We hypothesized that extremely late-sown winter wheat can attain high yield with an increased seeding rate, reduction in ET, and improved WUE by using only one irrigation at jointing and a suitable cultivar. The objectives of this study were to investigate soil water extraction (SWE), ET and biomass production at pre- and post-anthesis, and to determine a suitable irrigation regime and cultivar for high yield and WUE in extremely late-sown winter wheat under a high seeding rate in the NCP.

## Materials and Methods

### Site Description

Field experiments were conducted from November 2010 to June 2013 at the Wuqiao Experimental Station of China Agricultural University in Cangzhou, Heibei Province (37°41′ N, 116°37′ E). No specific permissions were required in the experimental site. The field studies did not involve endangered or protected species. This site is in the middle of the Heilonggang catchment in the northern part of the NCP. The altitude is 20 m above sea level. The underground water table was at 7–9 m. The soil was clay loam. In the 2 m soil profile, average bulk density was 1.55 g cm^−3^, average field capacity was 21.6% (g g^−1^), and wilting point was 7.6% (g g^−1^). The upper 40-cm soil profile contained 1.05% total organic matter, 0.08% total nitrogen, 14.93 mg kg^−1^ available phosphate, 107.20 mg kg^−1^ available potassium, and 46.10 mg kg^−1^ hydrolysable nitrogen. The station was in a temperate continental monsoon climate, characterized by dry, cold winters and rainy, hot summers. Weather data over 3 years in 2010–2013 were recorded at a meteorological station located at the experimental site (Table 1). During wheat growing season, total precipitation was 61 mm in 2010–2011 (2011), 148 mm in 2011–2012 (2012), and 130 mm in 2012–2013 (2013). Averaged temperatures in 2011, 2012, and 2013 growing seasons were 8.9C, 9.0C, and 7.9C, respectively. The seasonal sunshine hours were 1768 h in 2011, 1572 h in 2012, and 1429 h in 2013, as compared to the 30-year average of 1423 h.

**Table 1:** Monthly average air temperature, precipitation, and sunshine hours in the 2010–2011 (2011), 2011–2012 (2012) and 2012–2013 (2013) growing seasons.

Parameter	Nov.	Dec.	Jan.	Feb.	Mar.	Apr.	May.	Jun.	Mean/Total
Temperature (C)^a^									
2010–2011	5.9	0.6	-4.3	0.7	8.1	14.3	19.8	26.4	8.9
2011–2012	5.7	-1.2	-2.7	-0.5	6.4	16.0	22.2	25.9	9.0
2012–2013	4.3	-2.5	-4.0	-0.1	7.9	12.3	20.9	24.8	7.9
30-yr-avg.	6.3	-0.2	-2.1	1.4	7.4	15.1	20.8	24.8	9.2
Precipitation (mm)									
2010–2011	0	3	0	7	0	15	36	0	61
2011–2012	44	2	0	0	1	68	2	31	148
2012–2013	0	17	5	13	0	27	44	24	130
30-yr-avg.	11	4	3	7	11	20	40	20	116
Sunshine hour (hr)									
2010–2011	156	221	233	151	284	279	281	163	1768
2011–2012	104	157	127	215	218	246	305	198	1570
2012–2013	144	148	101	125	257	254	273	127	1429
30-yr-avg.	105	151	169	167	208	241	259	123	1423

^a^Monthly average air temperatures, precipitation, and sunshine duration in November and June were averages from sowing date to November 30 and from June 1 to harvest date, respectively.

### Experimental Design

The field experiment was designed as a split-plot experiment with irrigation regime as main plot and cultivar as subplot, with four replicates and a plot size of 4 m × 10 m. Two irrigation treatments included a one irrigation regime (W1, 75 mm at jointing) and two irrigation regimes (W2, 150 mm, 75 mm at jointing and 75 mm at anthesis). Water was supplied from a pump outlet to each plot through plastic pipes and a flow meter was used to measure the amount of water supplied. There was a 60-cm wide zone without irrigation between adjacent plots and an outer 100-cm wide zone without sampling in each plot to minimize the effects among different plots. 3 cultivars differing spike size were used in this study, including Hengshui4399 (HS4399, small spike), Jimai22 (JM22, medium spike), and Weimai8 (WM8, larger spike).

Winter wheat was sown on November 11, November 13, and November 15 and the corresponding seeding rates were 800, 850, and 800 seeds m^−2^ in 2010, 2011, and 2012, respectively. Preliminary field experiments demonstrated that winter wheat attained the highest spikes m^−2^ at the seeding rate of 800–850 seeds m^−2^. The seeding depth was 4–5 cm and row spacing was 15 cm. Over the 3 years, soil water before sowing in the upper 40-cm soil ranged from 75 to 80% of field capacity following a small irrigation (20–50 mm). A total of 225 kg N ha^−1^ as urea, 300 kg P ha^−1^ as ammonium monoacid phosphate, 150 kg K ha^−1^ as potassium sulfate, and 15 kg Zn ha^−1^ as zinc sulfate were broadcasted and incorporated into the upper 30 cm soil layer by rotary tillage prior to sowing. At the beginning of winter dormancy (early December, < 0C), wheat plant were at less than 1.0 Haun stage [5]. Seedlings greened up in early March after winter. Jointing stage occurred in mid-April and no tillers were developed. Wheat plants reached anthesis in mid-May and maturity around June 15. Both anthesis and maturity dates were two days earlier in HS4399 than in JM22 and WM8 in each growing season.

### Measurements

#### Crop Phenology

Crop phenology was recorded using the Zadoks scale [17] and main stem leaf stage was recorded using the Haun scale [5]. When 50% of plants reached jointing (Z31), anthesis (Z61), and maturity stage (Z91), corresponding dates were recorded and growing-degree days (GDD) after sowing were calculated. GDD during different growth periods were calculated as [18]:

$$GDD=\sum (\frac{T_{\text{max}}+T_{\text{min}}}{2}\;-\;T_{\text{b}})\;(\frac{T_{\text{max}}+T_{\text{min}}}{2}>T_{b})$$ (1)

where *T*_*max*_ and *T*_*min*_ were daily maximum and minimum air temperatures, respectively, and *T*_*b*_ was base temperature and defined as 0°C, following Kirby [19]. The results were expressed as °Cd.

#### Soil Water Extraction and Seasonal evapotranspiration

Soil water content measurements were made at sowing (Z00), anthesis (Z61), and maturity (Z91). Soil samples were collected from 0 to 200 cm at 20-cm intervals with a soil corer. Soil gravimetric water content (g water g^−1^ dry soil) was measured by oven-drying samples at 105°C for 48 h to constant weight. Soil volumetric water content (cm^−3^ cm^−3^) was determined by gravimetric water content (g water g^−1^ dry soil) and bulk density. Soil water extraction (SWE, mm) was calculated as the difference in stored soil water (0–200 cm) between two specific stages. Crop seasonal ET was calculated using the soil water balance equation [8] as:

ET=P+I+SWE−R−D+CR (2)

where ET (mm) is crop seasonal evapotranspiration, P (mm) is rainfall, I (mm) is irrigation, SWE (mm) is difference of water storage in the 200-cm soil profile between sowing and maturity, R is runoff, D is drainage below the 200 cm soil profile, and CR is capillary rise into the root zone. Because the groundwater table at the experimental site is 7–9 m (> 4 m) below the ground surface, CR is negligible. R and D can also be ignored in the NCP, including the experimental site [20].

#### Biomass and Morphological Traits

At anthesis and maturity, aboveground biomass was determined by sampling plants including roots in the upper 10-cm soil profile in two 100-cm inner rows of each plot. Thirty plants were randomly selected from each biomass sample at anthesis and maturity. Primary and secondary roots plant^−1^ were counted on each of 30 plants at anthesis, and the number m^−2^ was calculated by multiplying by spikes m^−2^ as described below, given that there were no tillers. Plant height and spike length were measured on each of 30 plants at maturity. Biomass samples after removal of roots were oven-dried at 80°C for 48 h to constant weight. For samples at maturity, grain weight was measured by threshing spikes followed by oven drying at 80°C for 24 h again. HI was calculated as the ratio of grain weight to total aboveground biomass at maturity. Post-anthesis biomass was calculated as the difference in biomass between maturity and anthesis. Biomass remobilization during grain filling (BMR) was calculated following the method of Xue et al. [21]:

BMR=BMA-(BM-grain biomass at maturity) (3)

where BMA (t ha^-1^) is biomass at anthesis, BM (t ha^-1^) is biomass at maturity.

#### Grain Yield and Water Use Efficiency for Biomass and Grain Yield

Spikes m^−2^ were counted in six 1-m central rows of each plot before harvest. The number of grains spike^−1^ was determined by counting the kernels in each spike from 60 randomly selected plants in each plot before harvest. At maturity, wheat plants from a 3-m^2^ area were harvested and threshed for grain yield determination. Actual grain yield was reported on a 13% moisture basis. TGW was calculated by weighing 1000 seeds in 3 replicates from each sample.

Water use efficiency for biomass (WUE_bm_) and grain yield (WUE) was calculated as follows [21]:

$${WUE}_{\text{bm}}=BM/ET$$ (4)

WUE=GY/ET (5)

where BM (t ha^-1^) is biomass at maturity; GY (t ha^-1^) is grain yield; ET (mm) is total seasonal evapotranspiration during period of wheat growth.

#### Statistical analysis

Analysis of variance (ANOVA) was conducted using the general linear model procedure in SAS [22] with appropriate error terms. Year, irrigation regime, cultivar, and their interactions were treated as fixed effects and replication as a random effect. The least significant differences at a probability level of 0.05 were calculated for mean comparisons. Means of two irrigation regimes and 3 cultivars were presented to describe irrigation and cultivar main effects, given that no irrigation × cultivar interaction was found for most variables.

## Results

### Crop Phenology

On average, days from sowing to jointing, anthesis, and maturity were 159, 184, and 216 d, respectively (Table 2). GDDs for the corresponding developmental stages were 576, 1040, and 1822°Cd, respectively. The differences in phenological stages among years were generally small when calendar days were used (< 5 days). However, the differences were large when GDDs were used, with the 2011 season having the highest and 2013 the lowest GDD from sowing to jointing. There was little variation in calendar days or GDDs among three years from jointing to anthesis and from anthesis to maturity. Cultivar HS4399 reached to anthesis and maturity 2 days earlier than JM22 and WM8. Similarly, GDD from jointing to anthesis and during the whole growing season was less for HS4399 than for JM22 and WM8 (Table 2).

**Table 2:** Days and growing-degree days (GDD) during different growing periods for three cultivars in the 2011, 2012, and 2013 growing seasons.

Season	Cultivar	Days (d)				GDD (°Cd)			
		Z00-Z31^a^	Z31-Z61	Z61-Z91	Total	Z00-Z31	Z31-Z61	Z61-Z91	Total
2011	HS4399	161	25	34	220	684	436	819	1939
	JM22	161	27	34	222	684	483	828	1995
	WM8	161	27	34	222	684	483	828	1995
	**Mean**	**161**	**26**	**34**	**221**	**684**	**467**	**825**	**1976**
2012	HS4399	160	21	32	213	548	432	796	1775
	JM22	160	23	32	215	548	470	778	1796
	WM8	160	23	32	215	548	470	778	1796
	**Mean**	**160**	**22**	**32**	**214**	**548**	**457**	**784**	**1789**
2013	HS4399	157	24	31	212	496	442	702	1639
	JM22	157	26	31	214	496	482	755	1733
	WM8	157	26	31	214	496	482	755	1733
	**Mean**	**157**	**25**	**31**	**213**	**496**	**469**	**738**	**1702**

^a^Z00, dry seed (sowing); Z31, first node detectable (jointing); Z61, beginning of anthesis (anthesis); Z91, caryopsis hard (maturity).

### Morphological Traits

The morphological traits of the 3 cultivars are shown in Table 3. Among the 3 cultivars, HS4399 had the smallest spike size with the lowest plant height, but WM8 had the largest spike size with the highest plant height, and JM22 has medium-sized spikes and plant height. As compared to WM8, HS4399 and JM22 had more primary roots but less secondary roots per plant or per square meter.

**Table 3:** Plant height (PH), spike length (SL) at maturity, primary and secondary root numbers for 3 cultivars across the 2011, 2012, and 2013 growing seasons.

Cultivar	PH	SL	Primary roots number		Secondary roots number	
	(cm)	(cm)	(plant^-1^)	(×10^3^ m^-2^)	(plant^-1^)	(×10^3^ m^-2^)
HS4399	68.3c	9.8c	5.4a	4.3a	9.9c	8.0b
JM22	71.5b	10.4b	5.1ab	3.9ab	10.3b	7.8b
WM8	76.0a	11.2a	5.0b	3.6b	13.5a	9.7a

Means followed by different letters are significantly different at the *P* < 0.05 level based on the least significant difference test.

### Soil Water Extraction (SWE)

**Fig 1:**
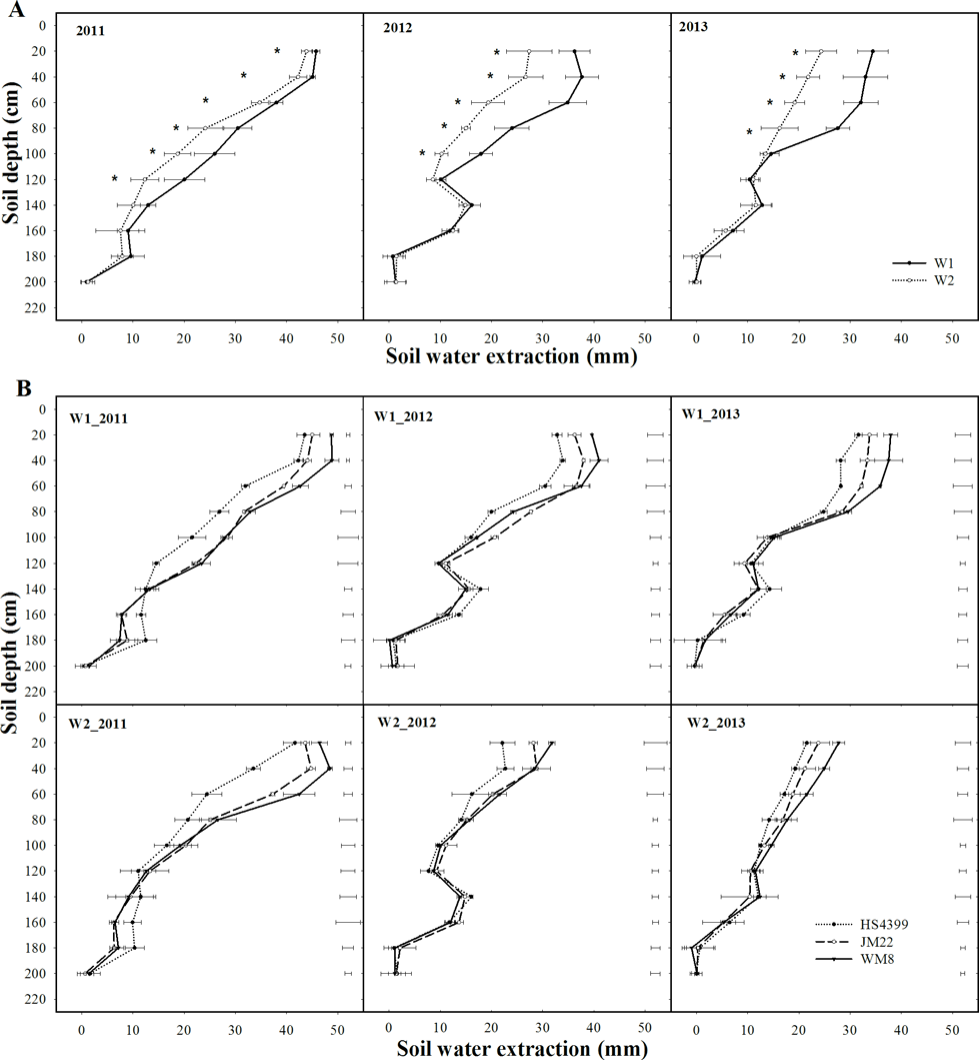
**Soil water extraction along the profile from sowing to maturity under two irrigation regimes across cultivars (A) and 3 cultivars under two irrigation regimes (B) in the 2011, 2012, and 2013 growing seasons.** Error bars represent standard deviations of the means; “*” represents significant difference at *P* < 0.05; vertical bars represent the least significant difference at *P* < 0.05.

Seasonal SWE was higher under W1 than under W2 in all 3 years, with average values of 201 mm and 156 mm over 3 years, respectively (Fig 1A and Table 4). For each irrigation regime, seasonal SWE occurred to a depth of 180 cm in dry year of 2011 and 160 cm in relatively wet years (2012 and 2013) (Fig 1A). The significantly (*P* < 0.05) higher seasonal SWE under W1 than W2 occurred in the 0–120, 0–100, and 0–80 cm soil layers in 2011, 2012, and 2013 during grain filling, respectively (Fig 1A and Table 4). Averaging irrigation regimes, cultivar JM22 had similar SWE to WM8 but more SWE than HS4399 in 2011 and 2012 (Table 4). WM8 extracted more soil water than JM22 and HS4399 in the upper soil layers in all 3 years. However, HS4399 and JM22 extracted more soil water than WM8 in the deeper soil layers under both W1 and W2 in the dry year of 2011 and under W1 in 2012 (Fig 1B). No significant differences in post-anthesis SWE were observed between JM22 and WM8 in 3 years (Table 4).

**Table 4:** Soil water extraction (SWE) and total evapotranspiration (ET) in different growth periods during the 2011, 2012, and 2013 growing seasons, as affected by irrigation, cultivar, and year.

Year	Treatment	SWE (mm)			ET (mm)		
		Z00-Z61	Z61-Z91	Z00-Z91	Z00-Z61	Z61-Z91	Z00-Z91
2011	Irrigation						
	W1	98a	140a	238a	232a	141b	374b
	W2	98a	105b	203b	232a	181a	414a
	Cultivar						
	HS4399	85b	114b	199b	219b	153b	373b
	JM22	102a	123ab	225a	236a	162ab	398a
	WM8	107a	130a	237a	241a	169a	410a
2012	Irrigation						
	W1	83a	121a	194a	275a	142b	417b
	W2	83a	60b	142b	275a	166a	441a
	Cultivar						
	HS4399	68b	87a	150b	261b	150a	411b
	JM22	86a	91a	172a	279a	154a	433a
	WM8	93a	94a	182a	285a	157a	442a
2013	Irrigation						
	W1	107a	78a	171a	240a	136b	375b
	W2	107a	21b	124b	240a	163a	403a
	Cultivar						
	HS4399	97b	48a	140b	234b	148a	382b
	JM22	100b	48a	144b	237b	149a	385b
	WM8	113a	52a	159a	249a	152a	401a

For each year in each column, means followed by different letters are significantly different at *P* < 0.05 based on the least significant difference test. Z00, Z61, and Z91 denote sowing, anthesis, and maturity, respectively.

### Evapotranspiration (ET)

Seasonal ET was affected (*P* < 0.02) by irrigation regime, cultivar, and year × irrigation interaction (Table 5). Averaging among cultivars, seasonal ET under W1 decreased by 11% in 2011, by 6% in 2012, and by 7% in 2013 as compared to W2, with average values of 389 mm (W1) and 419 mm (W2) over 3 years (Table 5). The lower seasonal ET under W1 was due to lower post-anthesis ET (Table 4). Among the 3 cultivars, JM22 had similar seasonal ET to WM8 but higher ET than HS4399 in 2011 and 2012. In 2013, JM22 had similar seasonal ET to HS4399 but lower ET than WM8 (Table 5). Similar trends were observed in pre-anthesis ET in 3 cultivars, but no significant differences were observed in post-anthesis ET among cultivars (Table 4).

**Table 5:** Wheat biomass at maturity, harvest index (HI), grain yield, spikes m^−2^, grains spike^−1^, grains m^−2^, 1000-grain weight (TGW), and water use efficiency for biomass (WUE_bm_) and grain yield (WUE), as affected by irrigation, cultivar, and year.

Year	Treatment	Biomass	HI	Grain yield	Spikes	Grains	Grains	TGW	WUE_bm_	WUE
		(t ha^-1^)		(t ha^-1^)	(no. m^-2^)	(no. spike^-1^)	(×10^4^ no. m^-2^)	(g)	(kg m^-3^)	(kg m^-3^)
2011	Irrigation									
	W1	15.36b	0.463a	7.41b	753a	31.77a	23.88a	34.53b	4.11a	1.99a
	W2	16.02a	0.467a	7.81a	746a	31.96a	23.77a	36.33a	3.87b	1.89b
	Cultivar									
	HS4399	14.70b	0.49a	7.54b	797a	29.53c	23.55a	34.00b	3.95a	2.03a
	JM22	15.86a	0.48b	7.93a	746ab	32.04b	23.91a	37.40a	3.99a	2.00a
	WM8	16.52a	0.43c	7.36b	705b	34.03a	24.01a	34.90b	4.03a	1.80b
2012	Irrigation									
	W1	15.83a	0.475a	7.82a	784a	31.30a	24.50b	34.03a	3.79a	1.88a
	W2	16.31a	0.469a	7.97a	807a	32.00a	25.76a	32.77b	3.70a	1.81a
	Cultivar									
	HS4399	14.66c	0.49a	7.50b	843a	29.24c	24.66a	31.75c	3.57b	1.83b
	JM22	16.10b	0.49a	8.29a	786ab	31.97b	25.13a	35.20a	3.72ab	1.92a
	WM8	17.45a	0.43b	7.89a	759b	33.75a	25.60a	33.25b	3.95a	1.78b
2013	Irrigation									
	W1	14.97a	0.445a	6.97a	739a	29.78a	21.98a	31.37a	3.98a	1.86a
	W2	15.25a	0.415b	6.55b	738a	30.79a	22.70a	29.43b	3.78b	1.62b
	Cultivar									
	HS4399	13.62c	0.45a	6.39c	771a	28.85b	22.25a	28.90c	3.57c	1.68b
	JM22	15.02b	0.45a	7.11a	743a	30.89ab	22.96a	31.75a	3.91b	1.85a
	WM8	16.68a	0.39b	6.78b	701a	31.12a	21.81a	30.55b	4.16a	1.69b
**Source of variance**										
Year (Y)		0.0002	<0.0001	<0.0001	0.0005	0.0394	<0.0001	<0.0001	0.0005	<0.0001
Irrigation (Irr)		0.0800	0.1407	0.7545	0.7925	0.2244	0.1481	0.3203	0.0237	0.0195
Cultivar (Cult)		<0.0001	<0.0001	<0.0001	<0.0001	<0.0001	0.6240	<0.0001	<0.0001	<0.0001
Y × Irr		0.6451	0.0070	<0.0001	0.4851	0.8210	0.3700	<0.0001	0.3888	0.0009
Y × Cult		0.1320	0.6870	0.0019	0.5759	0.5652	0.6612	0.5329	0.0139	0.0003
Cult × Irr		0.9470	0.7194	0.5644	0.4889	0.8176	0.2922	0.6010	0.9864	0.7252
Y × Cult × Irr		0.6336	0.5956	0.0862	0.9189	0.9666	0.7230	0.0225	0.8959	0.4855

For each year in each column, means followed by different letters are significantly different at *P* < 0.05 based on the least significant difference test.

### Biomass and Harvest Index

**Fig 2:**
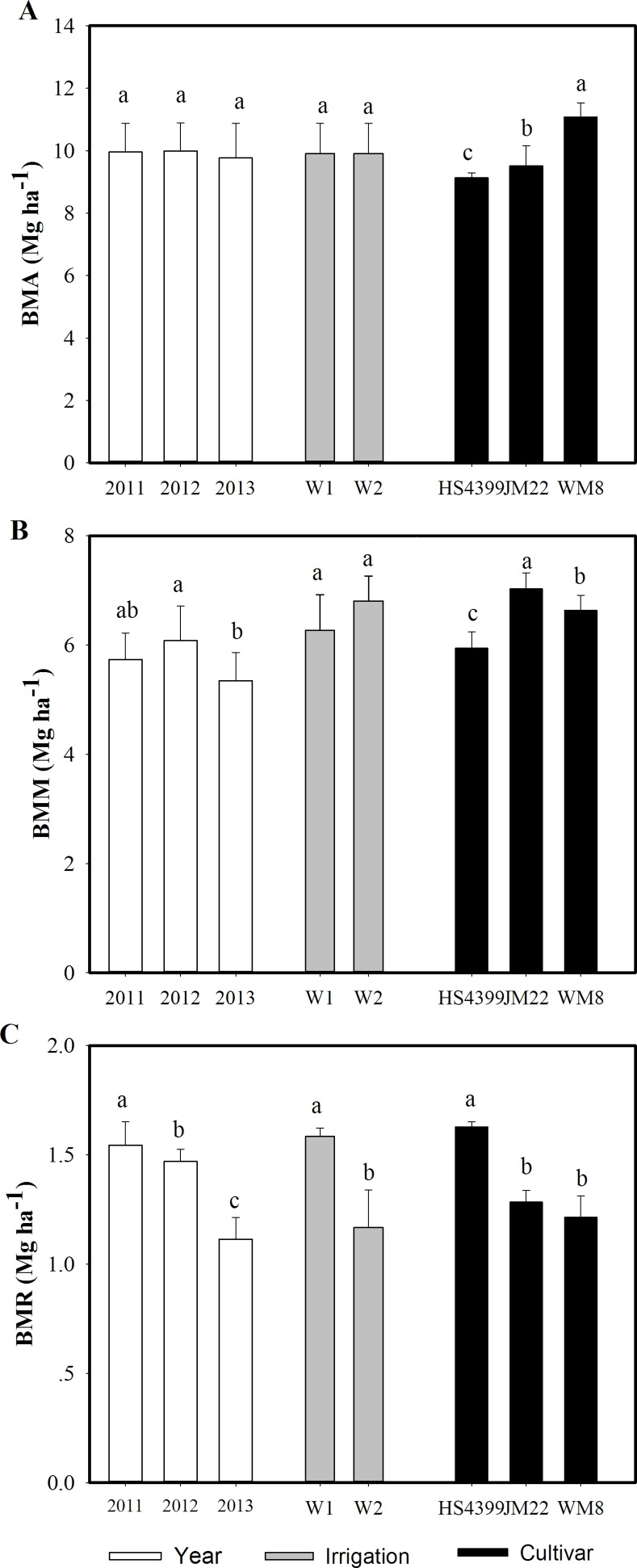
Biomass at anthesis (BMA), post-anthesis (BMM) and remobilization amount (BMR) during grain filling in 3 years (2011, 2012, and 2013), under two irrigation regimes (W1 and W2), and in 3 cultivars (HS4399, JM22, WM8). Vertical bars represent standard deviations of the means.

In general, there were no significant differences in biomass between two irrigation treatments (Table 5, Fig 2A and 2B). However, wheat under W1 had significant higher biomass remobilization (BMR) than that under W2 (Fig 2C). Among the 3 cultivars, WM8 had the highest biomass at maturity, followed by JM22, and HS4399 had the lowest (Table 5). Similar trends were observed in pre-anthesis biomass in 3 cultivars (Fig 2A). There was no significant difference in biomass between JM22 and WM8 in 2011. Meanwhile, JM22 had the highest post-anthesis biomass (BMM), which was 6.0% and 18% higher than WM8 and HS4399, respectively (Fig 2B). HS4399 had the highest BMR, 27% and 34% higher than BMRs of JM22 and WM8 (Fig 2C). As a result, the ratio of BMM to total biomass in JM22 and the contribution of BMR to grain biomass in HS4399 were the highest among the 3 cultivars.

The effect of irrigation on HI was associated with year, as evidenced by the significant (*P* < 0.01) year × irrigation interaction (Table 5). There was no difference in HI between irrigation treatments in 2011 and 2012. However, wheat under W1 had higher HI than that under W2 in 2013. Among the 3 cultivars, JM22 and HS4399 had higher HI than WM8 (Table 5).

### Grain Yield and Yield Component

Responses of grain yield and TGW to irrigation regime varied among the 3 years, owing to a significant (*P* < 0.001) year × irrigation interaction (Table 5). Compared to W2, grain yield and TGW under W1 were lower in 2011 but higher in 2013 (Table 5). Irrigation treatment had no effect on spikes m^−2^ and grains spike^−1^ (Table 5).

There was a significant (*P* < 0.05) cultivar effect on spikes m^−2^, grains spike^−1^, TGW, and grain yield (Table 5). HS4399 had the highest spikes m^−2^, followed by JM22, and WM8 had the lowest. In contrast, HS4399 had the lowest grains spike^−1^ and WM8 the highest. As a result, no grain m^−2^ differences were found among cultivars (Table 5). In all 3 years, JM22 had the highest yield and TGW, followed by WM8, and HS4399 had the lowest. The yield of JM22 was 6.0% and 9.0% higher than WM8 and HS4399 due to higher TGW, respectively. Grain yield in HS4399 was higher than WM8 in 2011 but lower in 2012 and 2013 (Table 5), resulting in a significant year by cultivar interaction at *P* < 0.05 (Table 5).

### Water Use Efficiency on Biomass and Grain Yield

There were significant differences (*P* < 0.05) in WUE_bm_ and WUE among years, irrigation regimes, cultivars, and interactions of year × irrigation and year × cultivar (Table 5). WUE_bm_ and WUE under W1 were higher than those under W2. Among the 3 cultivars, JM22 had the highest WUE in 3 years. HS499 had higher WUE than WM8 in 2011 but similar to WM8 in 2012 and 2013. On average, WUE in JM22 was 4.3% and 8.0% higher than HS4399 and WM8, respectively.

## Discussion

### Irrigation Regimes

The availability of soil water to crops depends on the root system [23]. In this study, wheat plants were able to extract soil water from as deep as 160–180 cm soil depth (Fig 1A), depending on the year. These results are similar to those of early sown wheat in the NCP (180–200 cm) [7, 24]. Plants under W1 had higher SWE and severe water stress was avoided under W1 during grain filling. No shorter grain-filling period was observed under W1 than under W2 in 3 years (data not shown). This was probably associated with greater water availability during grain filling because of decreased pre-anthesis ET at late sowing dates. The pre-anthesis ET in this study (average 249 mm) was less than result of 317 mm [25] in normal sown wheat at the same site. The decreased pre-anthesis ET resulted in the less seasonal ET of 389–419 mm as compared to 405–460 mm under W1 [8] and 457–482 mm under W2 [25] in normal sown wheat at the same site.

There was no yield loss under W1 in 2 of 3 years; even in the dry year of 2011, yield under W1 decreased only 5.3% but WUE increased by 5.0% than W2 (Table 5). The relatively stable yield was attributed to greater TGW and HI, which were associated with the higher BMR with similar post-anthesis biomass compared with W2 (Fig 2 and Table 5). The higher BMR was critical for W1 to maintain grain yield over 3 years. Early senescence under water stress leads to higher HI, grain weight, and yield under high nitrogen level [26]. In the present study, extremely late-sown wheat still had some green leaves even 5 days to maturity (data not shown), similar to the late maturity caused by high nitrogen levels. This probably explained why plants under W1 had higher grain weight in 2012. Moreover, late maturity increases the risk of hot, dry, windy weather. Studies showed that waterlogging after anthesis resulted in root injury and sudden leaf senescence [27, 28]. Substantial rainfall occurred in 2013 during mid-to-late grain filling (39 mm on May 26 and 24 mm on June 10). A shorter grain-filling period was observed under W2 than under W1 in 2013 (data not shown). These findings probably explained why plants under W1 had higher HI, kernel weight, and yield in 2013 (Table 5). This was similar to the results of over irrigation during late grain filling in previous studies [29, 30].

In the NCP, water use in wheat accounts for 70% of total water use in agricultural production [3]. Minimizing both water use and yield loss to maximize WUE under deficit irrigation has been one of top priorities for conserving groundwater resources. The WUE in this study was higher than the 1.57–1.75 kg m^−3^ [6] and 1.6–1.8 kg m^−3^ [8] values reported at the same site. This is consistent with the report that high WUE occurred with a balance of high yield and less ET [31]. Our result indicated that W1 reduced seasonal ET (374–417 mm) by 7.9% but enhanced WUE (1.86–1.99 kg m^−3^) by 7.5% due to stable yield compared with W2 over 3 years (Tables 4 and 5). These results suggested that one irrigation regime with extremely-late sowing was an important strategy to maintain sustainable agricultural development in the NCP.

### Cultivar Effects

Cultivar JM22 and HS4399 extracted more soil water than WM8 in the deeper soil layers under W1 and W2 in the dry year of 2011 and under W1 in a relatively wet year of 2012. Moreover, SWE decreased from upper to lower depths in the 0–100 cm soil layers among the 3 cultivars (Fig 1). These results indicated that WM8 faced a higher risk of water shortage in the upper soil layers. In contrast, cultivars JM22 and HS4399 were able to capture water from deeper soil layers under water stress. These results were in accord with the findings for root traits, with WM8 showing the most secondary roots and HS4399 and JM22 showing higher numbers and proportions of primary roots than WM8 (Table 3). Two studies of secondary root pruning have shown that fewer secondary roots decreased water extraction in upper layers and pre-anthesis ET, leading to lower seasonal ET [32, 33].

Our results indicated 5.9% and 8.9% higher yields over 3 years for cultivar JM22 than for WM8 and HS4399 (Table 5). Differences in yield among cultivars were associated primarily with TGW. Grain filling in wheat depends mainly on carbon from post-anthesis biomass and from remobilization of pre-anthesis biomass [34–36]. Two studies have found that decreased post-anthesis biomass under stress during grain filling generally reduced TGW, HI, and yield [30, 36]. Accordingly, the highest TGW, HI, and yield in JM22 were attributed to the highest post-anthesis biomass among the three cultivars (Fig 2 and Table 5). The positive correlation between wheat yield and post-anthesis biomass among cultivars has been reported in northern China [37, 38]. Feng et al. [39] reported that JM22 had high photosynthesis during grain filling even under heat stress. The high photosynthesis and post-anthesis biomass during grain filling, especially under heat stress (as in JM22), were critical for extremely late-sown winter wheat, as water availability was enhanced during grain filling owing to decreased pre-anthesis ET.

Our study indicated that JM22 had 4.2% and 9.3% higher WUE over 3 years than HS4399 and WM8, respectively (Table 5). The highest WUE in JM22 was associated with the highest yield, although JM22 had greater seasonal ET than HS4399. This was consistent with the result of Zhang et al. [3], who studied the genetic gains in yield and WUE and concluded that higher-yielding cultivars generally showed improved WUE. Condon et al. [40] summarized 3 key processes to improve WUE: preventing soil evaporation or extracting more soil water, improving WUE_bm_, and increasing HI. WM8 had the highest WUE_bm_ but the lowest WUE, owing to the lowest HI. HS4399 had the highest HI but the lowest WUE_bm_, leading to lower WUE than JM22. These results indicated that the 3 processes were not independent, with each negatively or positively affecting the other two. In this study, cultivar JM22 with a medium spike was shown to be most suitable for extremely late sowing at a high seeding rate in the NCP.

## Conclusion

Over 3 years, yields of 7.42 t ha^−1^ and WUE of 1.84 kg m^−3^ were achieved in extremely late winter sowing at a high seeding rate in the NCP. Plants under W1 treatment showed no lower yield in 2 of 3 years, with an average of 7.9% lower seasonal ET than W2, resulting in 7.5% significantly higher WUE than W2. The stable yield under W1 resulted from higher TGW and HI, associated mainly with higher SWE and BMR. Compared with HS4399 and WM8 across 3 years, the medium-spike cultivar JM22 achieved 5.9–8.9% higher yield using a seasonal ET falling within the range of the two cultivars, leading to 4.2–9.3% higher WUE. The higher yield for JM22 was a result of higher TGW and HI, due mainly to higher post-anthesis biomass and deeper seasonal SWE. These results showed that, in the NCP, one-irrigation at jointing can ensure grain yield benefit and maximize WUE in extremely late-sown winter wheat at a high seeding rate, especially for the medium-spike cultivar JM22.
